# Conceptual Framework for Implementation of Internationalization in Dental Education with Foundations in Dental Student Life

**DOI:** 10.3390/ijerph192013249

**Published:** 2022-10-14

**Authors:** Meghna Burad, Chanon Laowanichwith, Aimwadee Kiatsukasem, Sirirak Supa-amornkul, Kawin Sipiyaruk

**Affiliations:** 1 Mahidol International Dental School, Faculty of Dentistry, Mahidol University, Bangkok 10400, Thailand; 2 Department of Orthodontics, Faculty of Dentistry, Mahidol University, Bangkok 10400, Thailand

**Keywords:** academic performance, dental education, higher education, internationalization, extracurricular activity

## Abstract

The integration of internationalization within higher education has gained attention in both international and local programs, which allows the enrichment of the institutional quality. Previous literature reveals multidimensional considerations to determine the level of internationalization, considered as pre-existing performance indicators, including: (1) ‘Curriculum and academic offerings’; (2) ‘Collaboration and partnership’; (3) ‘Student and academic staff mobility’; (4) ‘Institutional policy’; (5) ‘Resources’; (6) ‘Campus life’; and (7) ‘Performance review and accountability’. This study aimed to investigate the impact of performance indicators of internationalization on academic performance and extracurricular activities among dental students. A validated online self-administered questionnaire was distributed to dental undergraduates. The data from 93 students (response rate: 96.86%) were analyzed using descriptive statistics and simple linear regressions. The findings demonstrated that all performance indicators appeared to have significant impact on self-perceived participations of extracurricular activities (*p* < 0.05), while only ‘Collaboration and partnership’ (*p* = 0.016), ‘Student and academic staff mobility’ (*p* = 0.009), ‘Institutional policy’ (*p* = 0.008), and ‘Campus life’ (*p* = 0.005) significantly affected actual participations. None of them appeared to be significant predictors for actual and self-perceived academic performance (*p* > 0.05). The statistical model constructed in this research can be utilized as a conceptual framework in the future establishment of internationalization among dental schools.

## 1. Introduction

With the growing prevalence of academic institutions interconnected through international collaborations, globalized perspectives and cosmopolitan influences, the concept of internationalization being amalgamated within higher education is captivating and quintessential [1]. Internationalization in higher education is constantly gaining prominence in institutional, national and international platforms [2]. The integration of internationalization within higher education can provide an institution with higher opportunities to enhance skills and knowledge for students, academic and support staff [3]. The goals to exhibit the global contributions of educational institutions could enhance the quality of educational institutions. 

The incorporation of internationalization within higher education, as in dental education, is advantageous in multiple aspects. From enhancing the socioeconomic capacity of countries, to amplifying cultural sensitivity and global knowledge, internationalization within higher education has the potential to enhance outcomes received by individuals and communities at different levels, ranging from students, to universities, to countries [3]. For nations, the positive aspects of internationalization range from the relocation of academic potential to the generation of profit from international students. For universities, benefits can be reaped through diversification of the learning environment [4]. As for individual students, qualities such as international mindedness and fluency in multiple languages are attractive for the job market [5]. These growing phenomena of internationalization are multidimensional, where the challenges, risks and benefits should be considered.

There appear to be a number of considerations to determine the internationalization of higher education institutions. ‘Curriculum and academic offerings’ appears to be the most important indicator concerning professional language courses, study abroad programs and academic staff responsible for developing the internationalized curriculum [6,7]. Student and academic staff mobility refers to students, staff and guest speakers that are capable of mobilizing to internationalized educational institutions [8]. ‘Collaboration and partnership’ inspects collaborative research, international networking, professional development, and teaching in cooperation with universities or institutions abroad [6,9]. ‘Institutional policy’ represents an institution’s internationalization commitments, efforts and its administrative leadership [6]. ‘Resources’ considers financial support, infrastructure, technological facilities and support from academic staff with sufficient competencies and language proficiency [10]. ‘Campus life’ scrutinizes the atmosphere created through student or staff-oriented organizations and various international and intercultural activities [6]. ‘Performance review and accountability’ measures international dimension qualities and achievements of the institution [11]. The combination of these performance indicators should be utilized to evaluate the level of internationalization.

Student life can be represented in a combination of curricular and non-curricular components [12]. As the curricular part is a set of instructions arranged as a part of a degree or a program, they can be reflected through academic performance [13]. The non-curricular component is a group of voluntary activities supported by programs or universities, which are usually assessed by the student participations of extracurricular activities [14]. According to the literature review above, the evidence implies that there could be the impact of internationalization on both curricular and non-curricular components. Therefore, with the absence of student life considerations, the establishment of internationalized higher education institutions cannot be comprehensively evaluated because the performance indicators of internationalization are mostly assessed by students.

International competition amongst higher education institutions to acquire global recognition appears to be increasing drastically, thus assessment of internationalization must be carried out thoroughly as it is a major benchmark in the process of university performance evaluation [6]. While the issue of internationalization has gained great attention in higher education, there is a scarcity of research assessing its impact on student aspects. Further research on internationalization in dental education should also be required [15]. Consequently, this research was conducted to investigate whether there were any performance indicators of internationalization that influence student life, inclusive of academic performance and extracurricular activities, within the undergraduate dental curriculum. This research would enhance our understanding in constructing a statistical framework demonstrating the impact of internationalization on dental student life. 

## 2. Materials and Methods

### 2.1. Research Participants

The population for this study was undergraduate students enrolled in Years 2–6 at the Mahidol International Dental School (MIDS), Faculty of Dentistry, Mahidol University. First-year students were not included due to their inadequate experiences in the program. According to the sample size calculation [16], the total population of 121 students was calculated for the expected number of participants in this research to achieve a 95% confidence level and 5% margin of error.

### 2.2. Questionnaire Design

This research study employed a quantitative survey design, using an online self-administered questionnaire (see Supplementary file) which was designed and distributed in Microsoft Forms. The questionnaire was constructed based on previous literature [8,9,10,11,17,18] with modifications to suit the research objectives. The questionnaire was divided into three sections which were: (1) demographics (5 items); (2) performance indicators of internationalization (35 items); and (3) self-perceived assessment on dental student life (12 items), including academic performance and extracurricular activities. Extracurricular activity involvement was also associated with stress and burnout impacting a student’s quality of life [19]. 

Prior to the distribution, the questionnaire was piloted and iteratively modified. The questionnaire content was firstly evaluated by three experts in dental and medical education where their comments afterwards were implemented for the revision until the index of item-objective congruence was higher than 0.5. The revised questionnaire was then piloted with Year 6 MIDS students in the previous academic year (*n* = 30), as they were not recruited as participants of this research, to ascertain the quality of the research instrument in terms of test–retest reliability (correlation coefficient higher than 0.7) and internal consistency (Cronbach’s alpha coefficient greater than 0.7). 

### 2.3. Student Database

Academic and extracurricular activity transcripts were retrieved from the student database with permission from MIDS. Cumulative Grade Point Average (cGPA) reported in the academic transcript and number of hours recorded in the extracurricular activity transcript were investigated to represent actual academic performance and extracurricular activity involvement, respectively. 

### 2.4. Data Analysis

The research data retrieved from the questionnaire and student database were analyzed using the Statistical Package for the Social Sciences (SPSS). Descriptive statistics were used to present data in overview. Simple linear regression analysis was performed to identify the significant impact of internationalization on dental student life, including: (1) self-perceived academic performance; (2) self-perceived participations of extracurricular activities; (3) actual academic performance; and (4) actual participations of extracurricular activities. The decision to use simple linear regression, rather than multiple linear regression, was based on the research objective to construct a statistical model. Statistical significance was taken at *p* < 0.05.

### 2.5. Ethical Considerations

The questionnaire was not anonymous, as its connection to the information retrieved from the student database was required. However, the research data were coded prior to the analysis. Consent forms had been obtained from all students who participated in this research. This research was approved by the Faculty of Dentistry and the Faculty of Pharmacy, Mahidol University, Institutional Review Board, reference number MU-DT/PY-IRB 2020/053.1109 on 11 September 2020.

## 3. Results

### 3.1. Research Participants

There were 93 dental undergraduates who provided their consent and completed the questionnaire (Table 1). A majority of participants were female and were studying in the third and fourth year of the six-year curriculum, which seemed to reflect the actual proportion of the populations. Most students were from local high schools, while only 12 participants completed their high school abroad. However, more than a half reported that they pursued their high school in international programs either in Thailand or in other countries. English appeared to be a home language in around a quarter of the participants. These represented mixed culture learning environments in Mahidol International Dental School.

### 3.2. Perceptions towards Performance Indicators of Internationalization

The participants tended to have slightly positive perceptions towards performance indicators of internationalization of their dental school (Table 2). ‘Performance review and accountability’ was perceived as the highest performance indicator with an average score of 3.67 (SD = 0.39) from 5, followed by ‘Collaboration and partnership’ (mean = 3.25, SD = 0.51) and ‘Student and staff mobility’ (mean = 3.11, SD = 0.56). Interestingly, ‘Campus life’ was rated by participants as the lowest performance indicator of their dental school with the score of 2.76 (SD = 0.70) from 5, where socializing spaces, extracurricular activities and cultural support were considered.

### 3.3. Assessments of Dental Student Life

The aspects of dental student life appeared to be perceived by participants as neutral, with the score of 3.09 (SD = 0.49) and 3.00 (SD = 0.58) for self-perceived academic performance and participations of extracurricular activities, respectively. The student database revealed that the mean of cGPA was 3.39 of 4.00 (SD = 0.34), representing actual academic performance. The data from the activity transcripts were also retrieved to demonstrate actual participations of extracurricular activities, where its mean was 26.53 h per year (SD = 12.52). These data are presented in Table 3.

### 3.4. The Impact of Performance Indicators on Dental Student Life

The results demonstrated significant impact of the performance indicators of internationalization on dental student life (Table 4). All of the indicators appeared to be significant predictors of self-perceived extracurricular activities, while most of the indicators of internationalization exhibited significant influence on self-perceived academic performance, which were ‘Collaboration and partnership’ (*p* = 0.016), ‘Student and academic staff mobility’ (*p* = 0.009), ‘Institutional policy’ (*p* = 0.008), and ‘Campus life’ (*p* = 0.005). Contrastingly, both self-perceived and actual academic performances appeared not to be significantly affected by performance indicators of internationalization. 

## 4. Discussion

Internationalization for higher education is defined as the integration of international and intercultural aspects into the institution’s goals, policies, and programs through its implementation within teaching and delivery of education [2,9,10,11,20]. The results from the simple regression model showed that all indicators of internationalization significantly impacted dental student life, supporting the conceptual framework of the seven performance indicators constructed using previous literature. Although a number of performance indicators of internationalization can affect the level of internationalization, this research found that none of them appeared to have significant impact on either self-perceived or actual academic performance. While all performance indicators seemed to significantly affect self-perceived participations of extracurricular activities, not all of them were found not to be significant predictors of actual participations of extracurricular activities.

Collaboration and partnership are required to fabricate a study abroad program, whereas student and academic staff mobility can facilitate international programs and increase student diversity [8]. Therefore, these two indicators can motivate students to participate in extracurricular events, where they can improve generic skills such as leadership, teamwork and self-motivation, and global skills like cultural awareness, networking, language, and communication skills [20]. Institutional partnerships as well as student and staff mobility are also considered as important components of internationalization in dental education [15]. There is evidence showing that dental students perceived an international peer learning setting as beneficial due to different perspectives in clinical principles and cultural exchange [21]. This research also found that ‘Curriculum and academic offerings’ had significant impact on self-perceived participations of extracurricular activities, but not on the actual participations. The extracurricular activity participation seems to be proportional to the benefits achieved from academics and the extent of academic involvement [22]. However, there was less influence of actual participations of extracurricular activities retrieved from the activity transcript, as students are required to participate in at least a minimum number of these events as a requirement of their graduation. 

To achieve internationalization, resources should include professional academic staff with sufficient competencies and language proficiency to enable activities to be conducted based on international standards [11]. Language skills can impact academic performance as they influence a student’s English proficiency and ability to comprehend content [23]. This, along with inadequate learning materials such as handouts and slides, lead to miscommunication and depression, as the inability to comprehend a language results in the accumulation of stress in the long term [24]. Although international staff recruitment and exchanges of dental academics can be a solution, there are a number of restrictions due to language and cultural concerns inclusive of license enabling clinical practice for the deliverance of patient care [25], which appears to be a major issue in several countries including Thailand. In addition, individual characteristics and campus environment can affect activity participation where the student life of an institution plays a significant role [26]. Students passionate about making a positive contribution to their university are more likely to be involved in extracurricular activities as they enable them to be an active part of the community whilst gaining knowledge and experience [27]. A faculty’s institutional policy significantly impacts extracurricular activity participation and generates constructive outcomes as frequent interaction amongst students and various members in a faculty positively influences a student’s satisfaction with their academic major and enhances career motivation [28]. Additionally, greater academic success and improved emotional wellbeing can be seen as a result of a student’s extracurricular activity participation [29]. 

Numerous factors comprising of an individual’s characteristics and the various components of a campus environment effect activity participation where the student life of an institution plays a significant role [26]. Students passionate about making a positive contribution to their university are more likely to be involved in extracurricular activities as they enable them to be an active part of the community whilst gaining knowledge and experiences [27]. Extracurricular activities can be considered as hidden curriculum to develop generic skills in dental students, preparing them to the realities of clinical practice [30]. The components of campus life such as the size of an institution proportionately determines the exposure and diversity the student has received in terms of events, activities, opportunities, and interactions with peers to enhance one’s social life. Such factors tend to influence both the actual and the self-perceived participation of students explaining the significance identified by the results of this study. 

Interestingly, performance indicators were found not to be significant predictors of self-perceived and actual academic performances. There is evidence that different learning style interventions in students have no impact on their performance [31]. Tailored learning styles for each student make no difference to their overall performance [32]. Even though unpreferred teaching styles are delivered, students will finally gain similar outcomes if the preferred teaching styles were given because they have to be responsible for their own education. Therefore, it can be implied that internationalization may not have a significant impact on student academic performance, due to the fact that most students acknowledged the need to take care of their own learning. They are likely to eventually search for ways to accomplish the best academic outcome on their own. However, the lack of internationalization should not be overlooked because teaching and learning style mismatch may require more time and effort from students to adapt and grow intellectually [33]. This would support students to learn with their convenience for the achievement of expected learning outcomes. 

**Figure 1:**
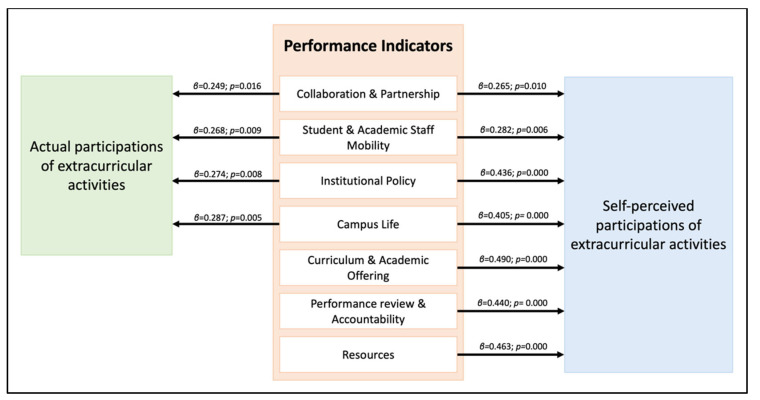
A statistical framework demonstrating the impact of internationalization on dental student life.

A statistical framework formulated from the results of this research demonstrates the impact of indicators of internationalization on dental student life (Figure 1). This framework makes it clearer for future establishment of an international dental curriculum to achieve the standard of internationalization by adapting this framework to the institution. Rather than a multiple linear regression, a simple linear regression was performed for this research, as its main objective was to investigate whether there were any performance indicators of internationalization on dental student life. The multicollinearity in a multiple regression model enabled only some variables to be expressed, demonstrating that some variables have a greater impact on the aspects of dental student life than others. Therefore, the simple regression model tends to demonstrate a greater number of influential factors than multiple regression.

Although this research was a single-site study, the data can be implemented into other similar contexts. However, further research in another setting should be required in order to ensure the transferability. Moreover, as the COVID-19 pandemic appears to have impacted on the quality of life of dental professionals [34], this issue should also be addressed to see if it has any impact on student life in international dental schools. In addition to academic performance and extracurricular activities, further research may investigate how performance indicators of internationalization can impact mental health, as it can be considered as essential for international students as they have higher expectations regarding international facilities and support, when compared to domestic students [35]. Furthermore, the justifications for the quantitative results should be further explained by in-depth qualitative data to deepen understanding on how students perceived those performance indicators, which will support higher education in the integration of internationalization.

## 5. Conclusions

The performance indicators of internationalization discussed in this research appeared to have impacted dental student life, especially self-perceived participations of extracurricular activities. Interestingly, there appeared to be no significant effect on academic performance, as students tend to adapt themselves to accomplish the best academic outcome. However, internationalization should still be taken into consideration, as it can affect the participation of extracurricular activities. The statistical framework constructed from the quantitative data can be implemented in the future for establishing international dental schools in order to ensure that programs abide by the standards of internationalization and cause no negative impact on dental student life.

## Figures and Tables

**Table 1:** Demographics of research participants.

Variables		*n*	%
Sex	Male	21	22.58
	Female	72	77.42
Academic Year	Year 2	18	19.35
	Year 3	25	26.88
	Year 4	26	27.96
	Year 5	17	18.28
	Year 6	7	7.53
High school sector	Local private school	64	68.82
	Local public school	17	18.28
	Other countries	12	12.90
High school curriculum	Local program	18	19.35
	International program	57	61.29
	Local program but conducted in English	18	19.35
Home language	Local language	70	75.27
	English	23	24.73

**Table 2:** Perceptions toward the seven performance indicators of internationalization.

Variables	Minimum	Maximum	Mean	SD
Performance review and accountability	2.40	4.00	3.67	0.39
Collaboration and partnership	1.80	4.00	3.25	0.51
Student and academic staff mobility	1.80	4.00	3.11	0.56
Curriculum and academic offerings	1.40	4.00	3.04	0.63
Institutional policy	1.60	4.00	3.03	0.54
Resources	1.00	4.00	2.98	0.62
Campus life	1.00	4.00	2.76	0.70

**Table 3:** Assessments of dental student life.

Variables	Minimum	Maximum	Mean	SD
**Self-perceived assessments of dental student life**				
Self-perceived academic performance	2.17	4.00	3.24	0.40
Self-perceived participations of extracurricular activities	1.33	4.00	3.00	0.58
**Actual assessments of dental student life**				
Actual academic performance (cGPA)	2.75	3.93	3.39	0.34
Actual participations of extracurricular activities per year (hours)	1.50	61.00	26.53	12.52

**Table 4:** Impact of performance indicators on dental student life.

Variables	Self-Perceived Academic Performance	Self-Perceived Participations of Extracurricular Activities	Actual Academic Performance (cGPA)	Actual Participations of Extracurricular Activities (Hours)
Performance review and accountability	β = 0.094 (*p* = 0.371)	β = 0.440 (*p* = 0.000)	β = −0.346 (*p* = 0.730)	β = 0.176 (*p* = 0.092)
Collaboration and partnership	β = 0.190 (*p* = 0.068)	β = 0.265 (*p* = 0.010)	β = 0.021 (*p* = 0.843)	β = 0.249 (*p* = 0.016)
Student and academic staff mobility	β = −0.158 (*p* = 0.130)	β = 0.282 (*p* = 0.006)	β = 0.042 (*p* = 0.687)	β = 0.268 (*p* = 0.009)
Curriculum and academic offerings	β = 0.018 (*p* = 0.863)	β = 0.490 (*p* = 0.000)	β = 0.106 (*p* = 0.311)	β = 0.162 (*p* = 0.121)
Institutional policy	β = 0.060 (*p* = 0.567)	β = 0.436 (*p* = 0.000)	β = 0.190 (*p* = 0.068)	β = 0.274 (*p* = 0.008)
Resources	β = 0.113 (*p* = 0.280)	β = 0.463 (*p* = 0.000)	β = −0.006 (*p* = 0.953)	β = 0.190 (*p* = 0.068)
Campus life	β = 0.015 (*p* = 0.886)	β = 0.405 (*p* = 0.000)	β = 0.000 (*p* = 0.999)	β = 0.287 (*p* = 0.005)

## Data Availability

The data that support the findings of this study are available from the corresponding author, upon reasonable request. The data are not publicly available due to information that could compromise the privacy of research participants.

## Supplementary Materials

The following supporting information can be available online at https://www.mdpi.com/article/10.3390/ijerph192013249/s1.
