# Supplementary material for: Conceptual Framework for Implementation of Internationalization in Dental Education with Foundations in Dental Student Life
Source: Int J Environ Res Public Health. 2022 Oct 14;19(20):13249. doi: 10.3390/ijerph192013249 (PMC9603489; doi:10.3390/ijerph192013249)
Supplement: Supplementary file 1 [file ijerph-19-13249-s001.zip › ijerph-1948596-supplementary.pdf]

Survey on perceptions towards the Internationalization of Mahidol dental school and impacts on student life

---

Part 1: Demographics

1. Student ID: ……………….

2. Sex
   Male
   Female

3. High school
   Public School in Thailand
   Private School in Thailand
   Abroad (please specify the country) ....

4. High school program
   Thai program
   International program
   Thai program but conducted in English

5. Preferred language of communication
   Thai
   English
   Other (please specify) ....

Part 2: Perceptions of internationalization

Rate each of the following items based on your perceptions towards the internationalization of your curriculum or faculty.

2.1 Curriculum and academic offerings

Items	Strongly disagree	Disagree	Neither agree nor disagree	Agree	Strongly agree
a. The curriculum has courses that enable students to have experiences abroad.					
b. The curriculum offers students with essential activities to create awareness of intercultural sensitivity, diversity and inclusivity as well as provides knowledge about other cultures.					
c. The curriculum provides students with opportunities to enroll in courses that enhance global perspectives such as global health, globalization etc.					
d. The curriculum provides students with optional foreign language courses.					
e. The curriculum includes international learning materials for students (e.g., case studies, articles written by authors from foreign countries).					

2.2 Collaboration and partnership

Items	Strongly disagree	Disagree	Neither agree nor disagree	Agree	Strongly agree
a. The faculty provides students and staff with activities that enhance international networking.					
b. The faculty has international research collaborations with other international institutions.					
c. The faculty is expanding international partnership with other universities/institutions.					
d. The faculty maintains quality within international partnerships.					
e. The faculty gives importance to international collaboration and partnerships.					

2.3 Student and academic staff mobility

Items	Strongly disagree	Disagree	Neither agree nor disagree	Agree	Strongly agree
a. The faculty/curriculum allows students to interact individually with foreigners such as guest presenters or lecturers from abroad.					
b. The faculty/curriculum gives its students a chance to interact with exchange students or students from abroad.					
c. The faculty/curriculum is committed to providing the students with educational experiences abroad.					
d. The faculty/curriculum supports students to experience other cultures through internships, exchange and study abroad programs.					
e. The faculty encourages the academic staff to get work experiences abroad.					

2.4 Institutional policy

Items	Strongly disagree	Disagree	Neither agree nor disagree	Agree	Strongly agree
a. The faculty considers internationalization to be a top institutional priority.					
b. The faculty ensures equal opportunity and benefit, amongst international and local students.					
c. The faculty has short-term and long-term plans to accomplish internationalization.					
d. The curriculum implements their strategies/policies to enhance internationalization. (e.g., teaching lectures in English, enrolling international students).					
e. The faculty/curriculum is committed to follow the plans for internationalization.					

2.5 Resources

Items	Strongly disagree	Disagree	Neither agree nor disagree	Agree	Strongly agree
a. The academic staff within the faculty are qualified to teach according to international standards.					
b. The academic staff within the faculty have sufficient competencies and language skills to teach in the international program.					
c. The support staff of the faculty have sufficient qualifications and language skills to assist in the international program.					
d. The faculty provides financial support to the student organization to carry out international activities within Thailand.					
e. The faculty provides prospective and current students with important information in English, through the website.					

2.6 Campus life

Items	Strongly disagree	Disagree	Neither agree nor disagree	Agree	Strongly agree
a. The faculty has meeting places that allow the local and international students to socialize.					
b. The faculty provides students with extracurricular activities that enhance their perspectives on internationalization.					
c. The faculty provides students with international and intercultural extracurricular activities on campus.					
d. The faculty offers diverse food options on campus for individuals with dietary restrictions (e.g., vegetarian food, halal food, etc.).					
e. The faculty provides students with international student support services (e.g., immigration assistance, prayer room or other religious/cultural facilities, etc.).					

2.7 Performance review and accountability

Items	Strongly disagree	Disagree	Neither agree nor disagree	Agree	Strongly agree
a. The faculty is a high-quality school.					
b. The faculty has positive reviews from social media platforms like Facebook and offline sources like newspapers or magazines.					
c. The faculty is a preferred institution for high school students to study dentistry.					
d. The faculty is a school with high prospects for future growth.					
e. The faculty is well-known through international rankings and awards.					

Part 3: Self-perceived assessment on dental student life

Rate each of the following items based on your perceptions towards your academic performance and extracurricular activities.

3.1 Academic performance

Items	Strongly disagree	Disagree	Neither agree nor disagree	Agree	Strongly agree
a. I find it difficult to understand the learning material provided.					
b. I am confident that I will pass all my exams for this academic year.					
c. It is important for me to obtain high grades for every course that I am enrolled in.					
d. I consider my academic performance to be above average.					
e. I think that I have adequate skills and abilities to continue this course and succeed in it.					
f. I am confident I will graduate within six years.					

3.2 Extracurricular activities

Items	Strongly disagree	Disagree	Neither agree nor disagree	Agree	Strongly agree
a. I would like to attend the extracurricular activities offered by the faculty.					
b. I think that the faculty provides beneficial and useful extracurricular activities.					
c. I feel that the faculty has various international activities to offer.					
d. I think that the extracurricular activities available at the faculty meet my needs.					
e. I believe that my activity experiences with the extracurricular activities are positive.					
f. I tend to invite my friends to attend the extracurricular activities offered by the faculty.					
